# Ten years Journal of Health Monitoring: Familiarity as a strength, openness as a responsibility

**DOI:** 10.25646/14462

**Published:** 2026-09-28

**Authors:** Oliver Razum, Joseph Kuhn, Dagmar Starke

**Affiliations:** 1 School of Public Health, Bielefeld University, Germany; 2 Pettenkofer School of Public Health Munich, c/o Bavarian Health and Food Safety Authority, Munich, Germany; 3 Academy of Public Health Services, Düsseldorf, Germany

There is no shortage of scientific journals, at least in quantitative terms. The Web of Science database indexes more than 22,000 journals in its Core Collection, whose journals are selected according to formal and scientific criteria. The SCImago ranking, based on data from the international bibliographic and citation database Scopus, lists more than 650 titles in the subject category *Public* *Health* *, Environmental and Occupational* *Health* alone. Our field is therefore well served by opportunities to publish. In this context, establishing a distinct scientific profile, a sustained readership, international recognition and a lasting place in scholarly exchange for a new journal is no easy task. The editorial team of the *Journal of* *Health* *Monitoring* has achieved precisely this. The journal has now been publishing for ten years. Since 2023, it has been listed in the *Directory of Open Access Journals*, a directory of open access journals assessed against quality criteria. Its English-language articles are deposited in the full-text archive *PubMed Central* and can be searched via *PubMed* and, since 2025, *Scopus*. This is an achievement that is as impressive as it is gratifying.

The* Journal of* *Health* *Monitoring* is published by the Robert Koch Institute (RKI). All articles appear in German and English. The journal thus serves two functions for the RKI: it provides international scientific visibility and is a key publication outlet for Federal Health Reporting in Germany. Its main target audiences include stakeholders in healthcare, policymaking and research. Health status, health behaviour, health determinants and healthcare structures are central themes of health reporting [1]. 

Many of the articles published in the journal examine health and socioeconomic differences between population groups, identify specific problems and needs, and document the social, structural and institutional conditions under which these differences arise. The journal thus supports efforts to reduce health inequalities in Germany. 

This is evident, for example, in its numerous articles on migration and health. Despite the diversity of the topics examined, these articles share the understanding that migration or ascribed origin alone cannot explain differentials in health. Rather, the decisive factors are living and working conditions, legal status and socioeconomic conditions, barriers to accessing healthcare, and experiences of exclusion and discrimination. Several articles in this field are among the journal’s frequently cited papers or have even become key references in the field. Examples include an empirical study of the health of children and adolescents with a migration history [2], a comprehensive review of the health and healthcare of asylum seekers and refugees in Germany [3], and a methodological paper providing recommendations for collecting and analysing data on migration-related determinants in public health research [4].

The* Journal of* *Health* *Monitoring* is publicly funded and does not charge publication fees. Its choice of topics therefore does not need to be guided by subscription revenue or the volume of fee-paying articles. Nor does the journal carry advertisements. This allows it to address topics concerning smaller population groups that may attract fewer citations. However, this strength also entails potential risks. First, cuts in public funding could affect the journal’s work in the future. Second, independence is an essential element of good health reporting [5]. Under future governments that might be influenced by right-wing populism, the independence the journal has enjoyed to date could be threatened, for example in its choice of topics. The history of the public health service in Germany shows that government health institutions are not immune to harmful political interference [6].

A further risk arises from a development that is positive in itself: many large datasets relevant to public health are now freely available worldwide. This makes data analysis easier and accessible to a larger number of researchers. However, increasingly powerful AI models also enable increasingly automated, large-scale generation of research questions, statistical analyses and manuscripts. This allows the mass production of formulaic publications with little scientific substance, or even misleading publications, by so-called paper mills: companies that produce scientific manuscripts to order [7].

Responsible topic selection and methodologically sound data analysis are therefore becoming ever more important. Here, the *Journal of* *Health* *Monitoring* benefits from the fact that many articles originate at the RKI or are produced in close collaboration with it. For these studies, the editorial team is particularly familiar with the datasets, how they were generated and the limits of the conclusions they support –and often also with the authors. This familiarity makes it easier to distinguish relevant research questions from analyses that are statistically possible but have little public health relevance.

An important challenge for the journal in the coming years will be to preserve the advantages of these close ties to data, topics and authors while avoiding a narrow, inward-looking institutional perspective. This includes systematically and continually involving external authors, drawing on other data sources and incorporating new and critical perspectives. In its first ten years, the *Journal of* *Health* *Monitoring* has done this remarkably well.
